# The trickle-down effect of predictability: Secondary task performance benefits from predictability in the primary task

**DOI:** 10.1371/journal.pone.0180573

**Published:** 2017-07-10

**Authors:** Magdalena Ewa Król, Michał Król

**Affiliations:** 1 Wrocław Faculty of Psychology, SWPS University of Social Sciences and Humanities in Wrocław, Wrocław, Poland; 2 Department of Economics, School of Social Sciences, University of Manchester, Manchester, United Kingdom; University of Würzburg, GERMANY

## Abstract

Predictions optimize processing by reducing attentional resources allocation to expected or predictable sensory data. Our study demonstrates that these saved processing resources can be then used on concurrent stimuli, and in consequence improve their processing and encoding. We illustrate this “trickle-down” effect with a dual task, where the primary task varied in terms of predictability. The primary task involved detection of a pre-specified symbol that appeared at some point of a short video of a dot moving along a random, semi-predictable or predictable trajectory. The concurrent secondary task involved memorization of photographs representing either emotionally neutral or non-neutral (social or threatening) content. Performance in the secondary task was measured by a memory test. We found that participants allocated more attention to unpredictable (random and semi-predictable) stimuli than to predictable stimuli. Additionally, when the stimuli in the primary task were more predictable, participants performed better in the secondary task, as evidenced by higher sensitivity in the memory test. Finally, social or threatening stimuli were allocated more “looking time” and a larger number of saccades than neutral stimuli. This effect was stronger for the threatening stimuli than social stimuli. Thus, predictability of environmental input is used in optimizing the allocation of attentional resources, which trickles-down and benefits the processing of concurrent stimuli.

## Introduction

The purpose of attention is to select information from the environmental input for further processing, based on the trade–off between maximization of information utility and minimization of costs related to information processing. This process of information selection is subject to a significant “budget constraint”, as the brain’s processing capacity is limited [1,2] and the metabolic cost of cortical computations is high [3]. Therefore, it would not be optimal to spend our limited processing capacity on information with low utility.

Thus, metaphorically speaking, attention is the gate-keeper of the mind—and given how important that role is, its methods of information selection must be of highest standard. There would be no point in investing in sophisticated methods of information processing if the information selected for processing at the beginning would be of low value. That of course leads to a paradoxical situation—the value of information must be assessed before it is processed. Therefore, there must be a set of rules that allow attention to optimally assess the value of information, but that do not require heavy processing.

As traditionally conceived, there are two sources of these rules: related to the properties of the stimulus (exogenous) and related to the current goals of the organism (endogenous). Exogenous (or bottom-up) attentional capture stems from the physical properties of the stimulus that are deemed important from the evolutionary point of view, i.e. which often signal the presence of valuable information. For example, salient stimuli are well known to capture attention, which allows for rapid orienting to potentially important features of the environment [4]. Stimulus features able to attract attention include abrupt onsets [5], unique properties [6], singletons [7], or novelty [8].

Moreover, certain classes of stimuli that are important from evolutionary point of view are also attended to preferentially and rapidly. Examples include threats [9], social stimuli such as faces [10] or human bodies [11], emotional [12] or sexual stimuli [13], animals [14] and animate motion [15].

In terms of endogenous (top-down) attentional control, attention is preferentially assigned to task-relevant stimuli [16,17]. This finding was also demonstrated in eye-tracking research [18]. Both exogenous and endogenous attentional capture have the same goal- the narrowing down of the environmental input to the most valuable information that are worthy of the expense of in-depth processing.

However, expectations constitute a separate class of processes that shape the selection of environmental information, and one that escapes the traditional dichotomy of endogenous vs. exogenous attentional capture [19–21].

According to the predictive coding approach to perception, expectations alleviate the processing burden by allowing for the distinction between expected and unexpected information [22]. The environmental data that conform to our expectations do not have to be processed in depth, as we already know them. That frees the processing resources, which in turn can be allocated to the processing of the unexpected. Expectations are of course created endogenously, but are not derived from our goals, tasks or motivations. Rather, they stem from our knowledge, experience and the representation of the regularities within the world that we learned [23]. Gibson [24] called this process the “education of attention”, that draws from the constancies and regularities of our environment, teaching us where to look and what to ignore.

Many studies demonstrate that expected stimuli are processed more efficiently, leading to more accurate responses and quicker detection. For example, presence of target stimulus in the expected area decreased search time [25], while previous exposure to a scene facilitated target detection [26]. Chun and Jiang [27] reported that detection of targets was accelerated when the configuration of distractors was predictive of the target location.

There is also plenty of evidence that unexpected or unpredictable events are attended to preferentially. For example, surprising elements in a scene attracted earlier fixations [28]. Moreover, surprising events attracted more gaze shifts than less surprising events [29].

In one sense, as Summerfield and Egner [19] observed, attention and expectation have a similar effect behaviourally—both facilitate detection and recognition. However, lack of precise distinction between these two, as Kok et al. [30] noted, leads to a confusion regarding the cognitive and neural effects of these processes. The crucial difference between attention and expectation lies in the amount of attentional resources assigned to an event expected in the sense of being a result of prediction, and an event expected in the sense of being awaited or goal-relevant (as a result of endogenous attention). The former would receive a smaller share of attentional resources, while the latter a larger one. For example, Kok et al. [30] reported that prediction leads to silencing of the sensory signal, but this effect can be reversed by attention, thereby demonstrating that these two processes have opposing effect on the sensory signal strength.

If predictable events require less processing resources, then there should be more spare resources available for other tasks. In other words, the amount of free resources that can be allocated to other current events will differ depending on the predictability of the primary event. As a result, the stimuli accompanying predictable stimuli should also be processed more effortlessly. This “trickle-down” effect is the essence of how expectations optimize processing. Silencing of the sensory signal related to predicted input decreases the processing workload, which should improve performance in concurrent tasks. For example, in dual task studies, experts perform better in secondary tasks than novices, because, due to their expertise in the primary task, they are able to allocate more attentional resources to the secondary task [31–33].

For this reason, dual task studies are an excellent method of exposing the limits of attention and the strategy behind the allocation of this scarce resource [34,35]. Another excellent measure of the allocation of attention (specifically visual attention) is the eye-movement analysis. According to Findlay and Gilchrist [36], vision is an active process, as we actively choose what we see via the eye movements. Attention and eye-movements are inseparably coupled [37,38] and because of that the allocation of attention is reflected in the eye-movements. In other words, eye movements reveal our strategies of selecting the information from the environment.

The purpose of this study was to demonstrate this mechanism in action using a dual task, where the primary task varied in terms of predictability. We wanted to test whether the predictability of the primary task will influence the attentional resource allocation between the two tasks. We hypothesized that (1) high predictability of the primary task will free some of the attentional resources and lead to an attentional shift to the secondary task, as reflected in eye-movement patterns. Additionally, we hypothesized that (2) this shift of attention will be accompanied by an improvement in the performance in the secondary task. However, we conjectured that (3) the allocation of attention based on predictability may be distorted with the appearance of a secondary stimulus with high evolutionary value, for example a threat or human face. Finally, we hypothesized that (4) participants who adjust to the predictability of the primary task to a higher extent will do better in the secondary task, in line with the results obtained in a previous study [39].

To this end, we used a similar task used in the Król, Kilan-Banach and Strzelecka [39] study. We asked the participants to perform two tasks simultaneously. The primary task involved dynamic stimuli (short video clips) representing a dot moving along one of three types of trajectories (random, semi-predictable and predictable). At some point during the clip, the dot transformed into a symbol for a short period of time. Participants were assigned a symbol beforehand and requested to press a key whenever their assigned symbol appeared.

The secondary task involved static stimuli- full colour photographs chosen from the International Affective Picture System [40]. In the threat block, half of the stimuli were threatening in content (representing violence, snakes etc.), while in the social block, half of the stimuli were social in content (containing at least one person). The other half of stimuli in both tasks were neutral emotionally and did not represent people. After finishing each experimental block, participants took part in an additional memory test. This checked their recollection of the static stimuli, by presenting some of the static stimuli displayed earlier in the experimental blocks, intermixed with similar but novel photographs. This task in its basic form has been tested in our previous study with a smaller sample, with only one type of static stimuli. The task we used previously also contained a confound, as both the moment of symbol appearance and the dot trajectory were manipulated in that experiment. That way, unfortunately, we could not say whether the experimental effect was due to the predictability of the dot trajectory or the time of symbol appearance. The experiment described here does not have this confound, as we manipulated only spatial predictability.

## Method

### Participants

Participants were 148 (105 females) volunteers, aged between 18–46 (*M* = 23.7; *SD* = 6). All participants had normal or corrected to normal eyesight. Participants were recruited for the study via the Faculty study advertisement system between October 2015 and April 2016 and took part in exchange for credits in the faculty credit system and/or 30 PLN (around 7 $) per hour. Of those, who responded to the advertisement, approximately 25% did not make or missed the appointment in the laboratory. The study was approved by the SWPS University of Social Sciences and Humanities, Faculty of Psychology II in Wrocław Research Ethics Committee, in accordance with the Declaration of Helsinki. Participants provided their written informed consent to take part in the study.

### Stimuli

**Fig 1:**
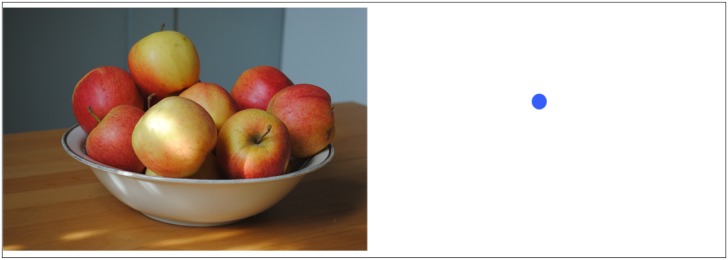
Example of a single frame within a trial. The original resolution of the image (fitting the whole screen) was changed for illustrative purposes. To the left, an image representative of the static stimuli (this photograph does not come from the IAPS, as IAPS photographs cannot be published. The author of this photograph is MEK). To the right, a single frame from the video clip (dynamic stimulus) showing the position of the dot.

Stimuli in the study were composites of two stimuli types: dynamic (short video clip) and static (a full colour photograph chosen from the IAPS [40], displayed simultaneously on the opposite sides of the screen (Fig 1).

Dynamic and static stimuli switched sides in the middle of the experiment and the initial positioning was randomized between participants.

#### Static stimuli

Static stimuli were full colour photographs selected from the IAPS, matched in terms of arousal and valence ratings provided by the authors of IAPS. Additionally, 17 participants (10 female, mean age = 26, SD = 4.7), who did not take part in the main study, provided ratings of visual complexity for all stimuli.

In the Social block, 27 non-neutral photographs representing one or more people were matched to 27 neutral photographs that did not contain people. There were no significant differences between neutral and non-neutral stimuli used in the Social block, in terms of valence (t(52) = 0.53, p = .60), arousal (t(52) = 0.16, p = .88) and complexity (t(52) = 0.55, p = .59). Therefore, these stimuli differed only in content, with non-neutral stimuli containing images of people, while neutral stimuli did not.

In the Threat block, 27 non-neutral stimuli had low valence ratings and high arousal ratings. They represented unpleasant, threating scenes, including violence, snarling dogs, snakes, and were matched to 27 neutral stimuli with neutral content. Neutral and non-neutral stimuli naturally significantly differed in terms of valence (t(52) = -17.26, p < .001) and arousal (t(52) = 7.17, p < .001). However, there was no significant difference in terms of complexity, t(52) = 0.63, p = .53 (for the mean ratings see Table 1 and for the detailed list of stimuli see S1 Table).

**Table 1:** Mean ratings of valence, arousal and complexity for neutral and non-neutral stimuli in each block (SD in parentheses).

Block	Valence	Arousal	Complexity
**Threat**			
**• Neutral**	6.59 (0.92)	4.55 (0.92)	5.10 (0.99)
**• Non-neutral**	2.86 (0.64)	6.17 (0.72)	5.27 (0.92)
**Social**			
**• Neutral**	6.13 (0.85)	3.86 (1.04)	5.08 (1.02)
**• Non-neutral**	6.27 (0.99)	3.98 (0.52)	5.22 (0.76)

Finally, 9 neutral photographs from each block were selected as targets in the memory test. They were matched with another two sets of 9 photographs (not displayed in the experimental blocks), that served as foils in the test and were also neutral in content. Thus, each of two sets of test stimuli contained 9 neutral targets (that also appeared in the preceding experimental block) and 9 neutral foils (that were not displayed in the experimental blocks). Targets and foils did not differ significantly in terms of valence, arousal and complexity.

#### Dynamic stimuli

**Fig 2:**
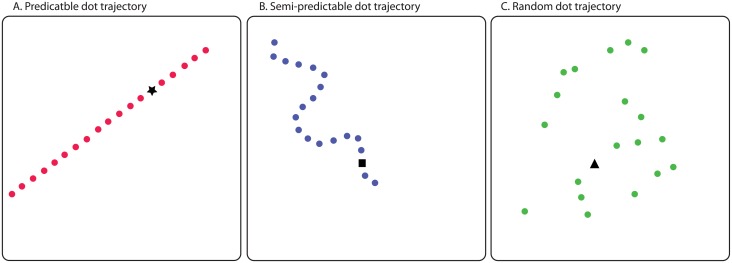
Examples of dot trajectories depending on their predictability. Predictable dot trajectory stayed the same throughout the study, while semi-predictable and random dot trajectories different from trial to trial. Only one dot was visible at a time.

Dynamic stimuli were animated clips generated using Mathematica (Wolfram), with a duration of 2600 ms. Each clip presented a dot moving on the screen along one of three possible trajectories. Dots following the predictable trajectory moved along a straight line that was identical in all clips in that condition (Fig 2a). Dots following the semi-predictable trajectory moved along an undulating but continuous line that was different for every clip in that condition (Fig 2b). Finally, dots following the random trajectory appeared in random places on the screen, where each subsequent position of the dot was independent of the previous one, and the sequence of dot positions was different for each clip in the condition (Fig 2c). The dots moved with the approximate speed of 10 cm per second.

Each type of trajectory was assigned to a different dot colour (red, green, blue), to give the participant the opportunity to learn predicting the trajectory based on the dot colour. Dot colour-to-trajectory assignment was randomized between participants.

At certain point during the clip, dots were replaced with a black symbol for 100 ms (star, triangle, square, diamond, and cross with equal probability). The change could happen at any moment between 1500–2000 ms. The mean onset of the symbol was identical in all conditions.

### Procedure

Participants’ eye movements were recorded using a remote eye-tracking device SMI RED250Mobile, with a sampling rate of 60 Hz and gaze position accuracy of 0.4°. Participants were seated 70 cm from the computer screen. The experiment was programmed in C# and displayed on a 15” Dell Precision M4800 workstation. Participants completed a 5-point calibration and 4 –point validation in-house procedure.

Participants were requested to pay attention to stimuli presented on both sides of the screen to the best of their ability. The study consisted of two blocks- the Social block and the Threat block, in an order randomized for each participant. Participants were informed that each block would be followed by a test of their memory of static stimuli displayed in the experimental block. They were also assigned one of the symbols at random and instructed to press a key whenever the moving dot transformed into their assigned symbol. This happened in 20% of all trials, determined at random, where the assigned symbols were distributed equally among the three dot trajectories. The memory test ensured attention to the static stimuli, while the assigned symbol detection task ensured attention to the dynamic stimuli. Dynamic and static stimuli switched their positions on the screen after the first block.

The study began with a training session consisting of 18 trials, including six of each type of dynamic stimuli (predictable, semi-predictable and random). These were accompanied by static stimuli, which would not appear later in the study. The results obtained in the training session were not analysed and training session was not followed by a memory test.

Each block consisted of three sessions, to give the participant the opportunity of a self-regulated break after each session. Each of the six experimental sessions comprised 18 trials, and included six trials of each type of dynamic stimuli and 9 trials of each type of static stimuli (neutral and non-neutral, equally distributed). Three of the eighteen static stimuli displayed in each session appeared again in the memory test as targets, while the rest were only displayed once. Each of the targets was accompanied by a different type of dot trajectory.

Trials started with a fixation cross displayed for 500 ms, followed by the static and dynamic stimuli composite displayed for 2600 ms, and a 500 ms blank screen.

The memory test followed each experimental block and consisted of 18 trials, with 9 targets (that were previously displayed in one of the preceding experimental sessions) and 9 foils (stimuli that were novel to the participants). Stimuli were displayed centrally on the screen for 3000 ms or until the key press indicating whether the participant remembered the stimulus (Yes/No). Thus, each participant saw 36 neutral pictures overall, half of these were new to them and half of them were displayed previously in the experiment. Of those, half (9) were displayed in the threat block, and the other half in the social block.

### Data analysis

#### Behavioural data

Before calculating signal detection measures, we applied a loglinear transform [41] in order to deal with extreme values of hits and false alarms (0 and 1), which were very common in the tasks. Criterion (*c*) was calculated as half of a standardized sum of proportions of hits and false alarms, that is:

$$c=-\frac{1}{2}\;\left[Z\left(hit\;rate\right)+Z\left(false\;alarm\;rate\right)\right]$$

while sensitivity (*d′*) was calculated as a standardized difference between the proportion of hits and false alarms, that is:

d′ = Z(hit rate) − Z(false alarm rate)

Z is the inverse of the normal distribution function.

#### Eye—Tracking data

In all analyses, we included only the eye-tracking data recorded during the duration of the stimulus (2700 ms.). Fixations and saccades were identified using the SMI Event Detector software, with minimum fixation length of 80 ms. We eliminated all trials in which the proportion of bad data samples (those in which the eye position could not be measured) was greater than 50% or in which no fixations were detected. There were six conditions (2 static stimuli types x 3 dot trajectories) in each of the two blocks (social and threat), so twelve conditions in total, with nine trials averaged for each condition. If there were no valid trials for any of the twelve condition, the whole dataset was eliminated, due to the repeated-measures design of the experiment. There were seven such cases (4.7% of all data). Additionally, we eliminated 8 (5.4% of all data) datasets where the proportion of fixations on any side of the screen was lower than 5%. This was either a results of low quality eye-tracking data obtained from that participant (due to calibration problems etc.) or because that particular participant did not follow the instructions or did not pay attention to the task. As a result, 133 datasets were used in all subsequent analyses.

We defined two Areas of Interest (AOI)—dynamic stimulus and static stimulus. Each was specified as covering the area equal to half of the screen divided vertically, that is 640 x 720 pixels (left side and right side, depending on which side the stimulus was displayed in a particular session). This amounted to 13.8° x 15.9° visual angle. Datasets accompanying this manuscript in the Supporting Information files contain only valid datasets.

## Results

### Behavioural data

#### Memory tests—Signal detection

We performed signal detection analysis separately for each memory test- after the social block (the social test) and after the threat block (the threat test).

Next, we performed the Wilcoxon signed-rank tests on both sensitivity and criterion. There was no significant difference between the social test and threat test in terms of either sensitivity, Z = -0.27, p = .79, or criterion Z = -.52, p = .61.

Additionally, mean sensitivity in the experiment was equal to 1.77 (SD = 0.62), which is significantly higher than chance level (d’ = 0), Z = -10.01, p < .001. Mean criterion in the experiment was significantly conservative (M = 0.31, SD = 0.29), Z = -8.74, p < .001, that is there was a tendency towards “No” responses.

#### Memory tests—Accuracy

It was impossible to obtain signal detection measures separately for the three levels of dot trajectory, because foils did not appear in the experimental blocks, and thus it was not possible to match them to specific dot trajectories. For this reason it was not possible to obtain the false alarm rates.

Foils in the memory tests were not paired with specific dot trajectories, but the target stimuli were. We were, therefore, able to analyze accuracy of responses to target stimuli in the test, depending on which dot trajectory they were accompanied with in the experimental sessions. Accuracy in the memory tests was calculated as the proportion of recognized old static stimuli to all old static stimuli displayed with that particular dot trajectory, i.e. hit rate in signal detection terms.

The experimental software drew at random nine of the static stimuli displayed in the experimental session to be displayed in the memory tests. As a result, the number of static stimuli accompanied by a specific type of ball trajectory that were displayed in the memory tests varied between participants. In case of sixteen subjects, for at least one dot trajectory no accompanying static stimuli were represented in the memory test, resulting in missing data. These subjects were excluded from the current analysis, leaving a sample of 116 subjects. All target stimuli were neutral, so stimulus type (neutral vs non-neutral) factor was not taken into account. Signal detection analysis described in the previous section showed no significant differences between the blocks, so for this reason we collapsed accuracy rates across the two blocks.

**Fig 3:**
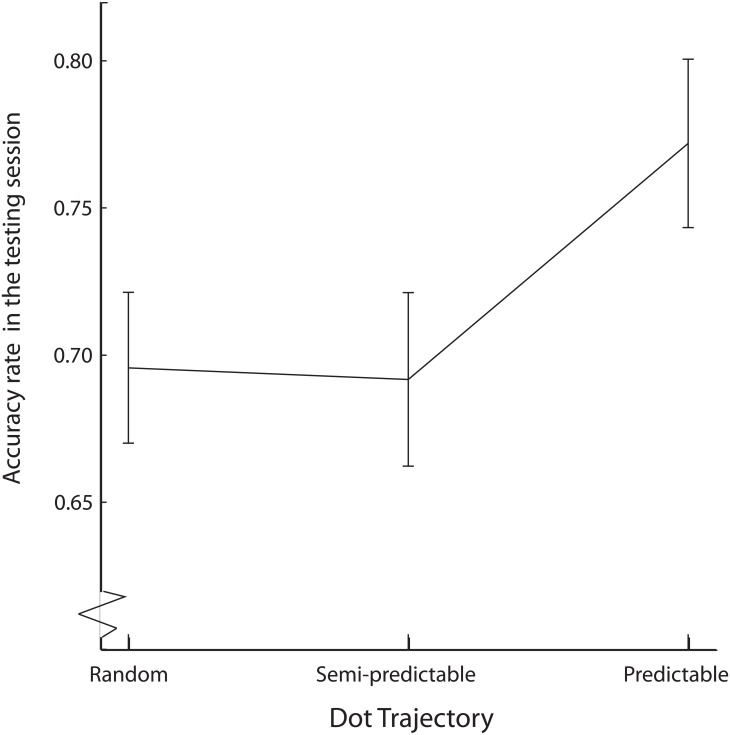
Accuracy rates in the testing session, depending on the dot trajectory accompanying the static stimulus. Accuracy is collapsed across tasks. Error bars denote within-subjects 95% confidence intervals.

We performed Friedman’s test on accuracy levels for all three levels of dot trajectory. The test was followed with post-hoc analysis using Bonferroni-corrected Wilcoxon signed-rank tests. Post-hoc tests were performed in the fashion of repeated contrasts, resulting in two tests and a corrected alpha level of 0.025. There was a significant main effect of dot trajectory on accuracy in the memory test, χ^2^(2) = 10.90, p < .01 (Fig 3).

Post-hoc analysis revealed that static stimuli accompanied by the dot moving with predictable trajectory were recognized more accurately in the memory test, compared to the static stimuli that were accompanied by the semi-predictable dot, Z = -2.28, p = .02. There was no significant difference between static stimuli accompanied by semi-predictable and random dots, Z = -0.23, p = .82.

#### Detection of assigned symbols—Signal detection analysis

The purpose of this task was simply to ensure attention to the dynamic stimuli. The appearance of the assigned stimuli was a rare event that was determined at random. As a result, for some participants the assigned symbols did not appear for every combination of experimental variables. For this reason, we collapsed the block and stimulus type factors, calculating accuracy only for the three levels of dot trajectory. However, preliminary analyses we performed before collapsing across these conditions indicated that neither block nor stimulus type were likely to have a significant effect on detection of symbols.

Mean sensitivity for the detection of signals was equal to 1.51 (SD = 0.89), which is significantly higher than chance level (d’ = 0), Z = -9.47, p < .001. Mean criterion for the detection of symbols was significantly conservative (M = 0.23, SD = 0.50), Z = -5.14, p < .001.

Due to non-parametric nature of the data, we performed Friedman’s test on both sensitivity and criterion for the three types of dot trajectories (random, semi-predictable and predictable). The test was followed with post-hoc analysis using Bonferroni-corrected Wilcoxon signed-rank tests, with corrected alpha level of 0.017.

In case of sensitivity, the main effect of dot trajectory was significant, χ^2^(2) = 9.37, p = .01. Post-hoc tests revealed that random dot trajectory was related to significantly lower sensitivity than both semi-predictable, Z = -2.60, p = .01, and predictable dot trajectories, Z = -3.32, p = .01. There was no significant difference between predictable and semi-predictable trajectories, Z = -0.52, p = .61.

In case of criterion, the main effect of dot trajectory was significant, χ^2^(2) = 56.46, p < .001. Post-hoc tests revealed that criterion was more conservative in the random dot trajectory condition than in both the predictable, Z = -5.49, p < .001, and semi-predictable condition, Z = -5.98, p < .001. There was no significant difference between the predictable and semi-predictable conditions, Z = -0.88, p = .38 (for a figure, see S1 Fig).

### Eye-tracking data

#### Proportion of time spent looking at the dynamic stimulus

In order to get a fair measure of the visual attention allocation, we calculated the total time spent looking at each AOI in each trial (time spent looking on the dynamic stimulus henceforth). In order to do that, the whole stimulus-related eye data sample was processed instead of single fixations, i.e. a sequence of 60 pairs of X-Y coordinates per second (given the 60 Hz sampling rate), one for each eye. This measure allowed us to take into account all types of eye-movement activity that may be taking place, and that escape the definition of a fixation. This may give a more accurate picture of eye-movement activity for the dynamic stimulus, where motion is involved and fixations may not be the best measure to capture the entirety of eye-movement activity in response to these stimuli. However, we have also performed an analysis involving the duration of fixations, which yielded similar results and is available in S1 Appendix.

**Fig 4:**
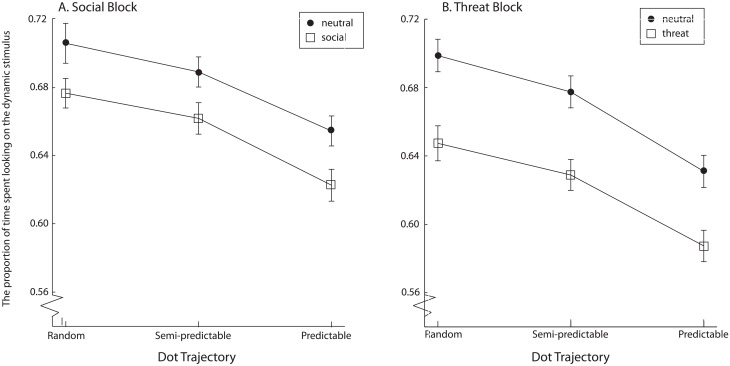
The proportion of time spent looking on the dynamic stimulus, separately for the neutral and non-neutral (social or threat, depending on the task) stimuli. Error bars denote within-subjects 95% confidence intervals.

We subjected the time spent looking at the dynamic stimulus to a repeated-measures 2 (block: social vs. threat) x 2 (static stimulus type: neutral vs. non-neutral (either social or threatening, depending on the block) x 3 (dot trajectory: random, semi-predictable, predictable) ANOVA (Fig 4)

Participants spent more time looking at the dynamic stimulus in the social block than in the threat block, F(1,132) = 3.92, p = .05, η_p_^2^ = .03. Participants spent more time looking at the dynamic stimulus when the accompanying static stimulus was neutral, than when it was not neutral, F(1,132) = 135.90, p < .001, η_p_^2^ = .51. Finally, there was a significant difference in the time spent looking on the dynamic stimulus depending on the dot trajectory, F(1,132) = 92.28, p < .001, η_p_^2^ = .41. Contrasts revealed that participants looked significantly less at the dynamic stimulus when the dot trajectory was predictable compared to when it was semi-predictable, F(1,132) = 99.23, p < .001, η_p_^2^ = .43. Contrasts also revealed that they looked significantly longer at random dynamic stimuli than semi-predictable ones, F(1,132) = 14.21, p < .001, η_p_^2^ = .10.

There was a significant interaction between block and stimulus type, F(1,132) = 9.54, p = .01 η_p_^2^ = .07, implying that the difference between the neutral and non-neutral stimuli was larger in the threat block, compared to the social block. The interaction between block and dot trajectory was not significant, F(2,264) = 1.24, p = .29, η_p_^2^ = .01, and neither was the interaction between stimulus type and dot trajectory, F(2,264) = 0.09, p = .92, η_p_^2^ < .01. Finally, the three-way interaction between block, stimulus type and dot trajectory was also non-significant, F(2,264) = 0.24, p = .79, η_p_^2^ < .01.

#### Proportion of the number of saccades to the dynamic stimulus

This measure was calculated in a similar manner to the proportion of duration of fixations- i.e. we calculated the proportion by dividing the number of saccades to the dynamic stimulus by the total number of saccades.

**Fig 5:**
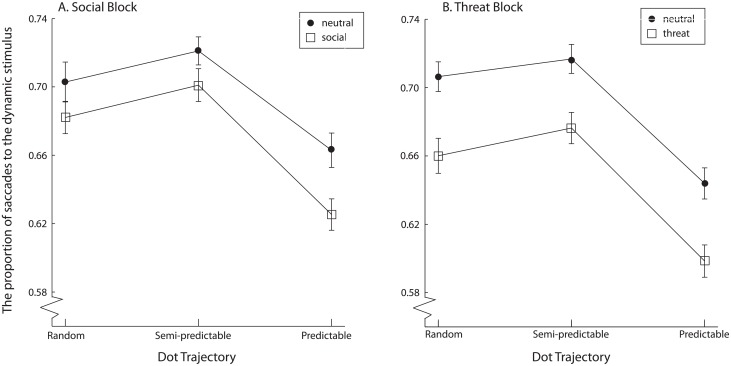
The proportion of saccades to the dynamic stimulus, separately for the neutral and non-neutral (social or threat, depending on the task) stimuli. Error bars denote within-subjects 95% confidence intervals.

We subjected the proportion of the number of saccades to the dynamic stimulus to a repeated-measures 2 (block: social vs. threat) x 2 (static stimulus type: neutral vs. non-neutral (either social or threatening, depending on the block) x 3 (dot trajectory: random, semi-predictable, predictable) ANOVA (Fig 5).

The difference between the proportion of number of saccades to the dynamic stimulus in the social block and in the threat block was insignificant, F(1,132) = 3.53, p = .06, η_p_^2^ = .03. The number of saccades to the dynamic stimulus was higher when the accompanying static stimulus was neutral, than when it was not neutral, F(1,132) = 122.40, p < .001, η_p_^2^ = .48. Finally, there was a significant difference in the proportion of the number of saccades to the dynamic stimulus depending on the dot trajectory, F(1,132) = 148.70, p < .001, η_p_^2^ = .53. Contrasts revealed that participants significantly less saccades to the dynamic stimulus when the dot trajectory was predictable compared to when it was random, F(1,132) = 138.70, p < .001, η_p_^2^ = .51. Contrasts also revealed that there were significantly more saccades to the dynamic stimulus in the semi-predictable condition, compared to the random condition, F(1,132) = 13.65, p<0.001, η_p_^2^ = .09.

There was a significant interaction between block and stimulus type, F(1,132) = 9.89, p < .01, η_p_^2^ = .07, implying that the difference between the neutral and non-neutral stimuli was larger in the threat block, compared to the social block.

The interaction between block and dot trajectory was not significant, F(2,264) = 1.68, p = .19, η_p_^2^ = .01, and neither was the interaction between stimulus type and dot trajectory, F(2,264) = 1.41, p = .25, η_p_^2^ = .01. Finally, the three-way interaction between block, stimulus type and dot trajectory was also non-significant, F(2,266) = 0.97, p = .38, η_p_^2^ < .01.

#### Analysis of different types of saccades

In order to obtain a more fine-grained analysis of saccades, we divided all saccades into three types: saccades within the static stimulus (both starting and ending within the static stimulus AOI), saccades within the dynamic stimulus (both starting and ending within the dynamic stimulus AOI), and saccades between the two types of stimuli (starting and ending in a different AOI). Next, we performed a repeated-measures 3 (saccade type: within static, within dynamic, between static and dynamic) x 2 (static stimulus type: neutral vs. non-neutral) x 3 (dot trajectory: random, semi-predictable, predictable) ANOVA on the number of saccades. Greenhouse- Geisser correction was used to adjust the degrees of freedom, when the sphericity assumption was violated. There was a significant main effect of saccade type, F(1.21, 159.81) = 383.88, p < .001, η_p_^2^ = .74. Contrasts revealed that there were significantly fewer saccades within the static AOI (M = 1.41, SD = 0.08) than between the AOIs (M = 1.86, SD = 0.05), F(1,132) = 47.94, p < .001, η_p_^2^ = .27. Contrasts also revealed that there were significantly fewer saccades between the AOIs (M = 1.86, SD = 0.05), than within the dynamic AOI (M = 5.16, SD = 0.16), F(1,132) = 365.90, p < .001, η_p_^2^ = .74.

There was no significant difference between neutral and non-neutral stimuli, F(1,132) = 0.04, p = .85, η_p_^2^ < .01, but the main effect of dot predictability was significant, F(2,264) = 73.14, p < .001, η_p_^2^ = .60. The interaction between the stimulus type and saccade type was non-significant, F(1.24, 164.2) = 2.26, p = .11, η_p_^2^ = .02, and so was the interaction between the stimulus type and dot predictability, F(2,264) = 0.25, p = .78, η_p_^2^ < .01. However, the interaction between the saccade type and the dot predictability reached significance, F(2.44, 321.88) = 182.95, p < .001, η_p_^2^ = .58. Finally, the three-way interaction between stimulus type, saccade type and dot predictability was insignificant, F(2.66, 350.67) = 0.10, p = .98, η_p_^2^ < .01.

Given that the interaction between saccade type and dot trajectory was significant, we run three follow-up ANOVAs with dot predictability as the factor. We collapsed the stimulus type variable given there was neither a significant main effect nor a significant interaction in the omnibus ANOVA.

The effect of dot predictability was significant for all three saccade types: within the static AOI: F(1.88, 248.75) = 72.18, p < .001, η_p_^2^ = .35, within the dynamic AOI: F(2,264) = 201.93, p < .001, η_p_^2^ = .61, and between the AOIs: F(1.91, 252.60) = 155.56, p < .001, η_p_^2^ = .54.

**Fig 6:**
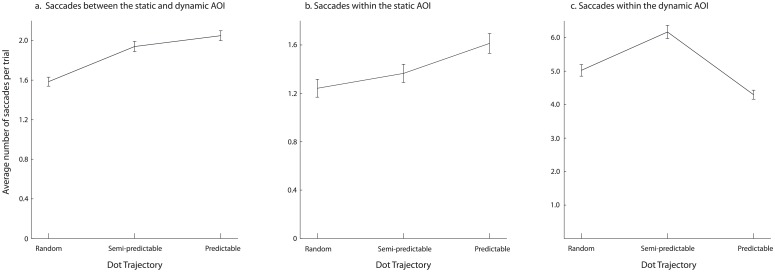
The number of saccades a. between the AOIs, b. within the static AOI and c. within the dynamic AOI, depending on the dot trajectory predictability. Note that for the clarity of presentation of results each diagram has a different scale on the y-axis.

In case of the saccades between the AOIs, contrasts revealed that there were significantly fewer saccades in the random dot condition, compared to the semi-predictable dot condition, F(1,132) = 257.57, p < .001, η_p_^2^ = .66, and significantly fewer saccades in the semi-predictable dot condition compared to the predictable dot condition, F(1,132) = 19.85, p < .001, η_p_^2^ = .13 (Fig 6a).

In case of the saccades within the static AOIs, contrasts revealed that there were significantly fewer saccades in the random dot condition, compared to the semi-predictable dot condition, F(1,132) = 122.75, p < .001, η_p_^2^ = .48, and significantly fewer saccades in the semi-predictable dot condition compared to the predictable dot condition, F(1,132) = 82.50, p < .001, η_p_^2^ = .39 (Fig 6b).

In case of the saccades within the dynamic AOI, contrasts revealed that there were significantly fewer saccades in the random dot condition, compared to the semi-predictable dot condition, F(1,132) = 57.42, p < .001, η_p_^2^ = .30, but significantly more saccades in the semi-predictable dot condition compared to the predictable dot condition, F(1,132) = 419.17, p < .001, η_p_^2^ = .76 (Fig 6c). Finally, for a distribution of saccade length, depending on dot trajectory predictability, please see S2 Fig.

### Eye-movement correlates of accuracy

First, we wanted to evaluate whether the ability to remember the static stimulus was dependent on the amount of time participants spent looking at that particular stimulus when it was first presented in the experimental block. For each participant and for each static stimulus that was presented in both the experimental block and the memory test, we calculated the average distance between the center of that stimulus in the experimental block and the position of the eyegaze throughout that trial. Next, we performed a rank biserial correlation between the average distance between the eyegaze and the center of the stimulus at first presentation, and remembering it in the second presentation, i.e. in the memory test. We found that larger distance between the center of the stimulus and the eyegaze at first presentation was negatively correlated with remembering the stimulus in the memory test, r_s_ = -.20, p < .001.

There was also a significant negative correlation between the mean proportion of time spent looking at the dot and sensitivity in the memory test, r_s_ = -.22, p = .01.

Finally, we defined “predictability adjustment” as the participants’ ability to change the allocation of their looking time depending on the predictability level of the dynamic stimulus. More specifically, it was calculated as proportion of time spent looking at the dynamic stimulus with random trajectory divided by the proportion of time spent looking at the dynamic stimulus with predictable trajectory- higher values signified larger difference in looking time between the two conditions and thus, higher “predictability adjustment”.

There was no significant correlation across participants between predictability adjustment and sensitivity in the memory tests, r_s_ = .03, p = .72.

## Discussion

The main goal of the study was to investigate whether predictability of the primary stimulus leads to a reduction in attentional resources allocation to that stimulus and, as a consequence, frees the resources for the processing of concurrent stimuli. This in turn should lead to more in-depth processing of the secondary stimulus, reflected in more robust encoding and better future recollection of that stimulus. This hypothesis was operationalized using a dual-task and testing the influence of stimulus predictability in the primary task on the attention allocation and performance in both tasks. Additionally, we wanted to test whether stimuli particularly important from evolutionary point of view, will be capable of distorting the allocation of attention driven by stimulus predictability. We used eye-movement patterns as the measure of visual attention allocation. We also performed signal detection analysis (where possible) and analysed the accuracy of responses in both primary and secondary tasks.

Summarizing the results, in support of our first hypothesis, we found that participants allocated more attention to unpredictable (random and semi-predictable) stimuli than to predictable stimuli. We also found evidence in favour of the second hypothesis, as participants performed better in the secondary task (as evidenced by higher sensitivity), when the stimuli in the primary task were more predictable. Our third hypothesis was supported by the finding that “special” stimuli (social or threatening) were allocated with more “looking time” than neutral stimuli. This effect was stronger for the threatening stimuli than social stimuli. Additionally, the number of saccades to the dynamic stimulus was smaller, when the static stimulus was “special”- either social or threatening.

Additionally, when the stimuli in the primary task were more predictable, participants performed better in the secondary task, as evidenced by higher sensitivity in the memory test.

However, we found no evidence for our final hypothesis, that the ability to adjust attentional allocation to stimulus predictability will be related to better performance in the secondary task. We found no correlation between predictability adjustment- the extent to which participants changed their looking patterns depending on dynamic stimulus predictability, and performance in the memory task.

### The effect of predictability on eye-tracking and behavioural measures

We found a strong effect of predictability on all eye-tracking measures and most importantly on performance in the memory test. Time spent looking at the dynamic stimulus was larger for the unpredictable (random and semi-predictable) dot trajectories compared to the predictable trajectory. Similar effect was obtained also for the proportional duration of fixations within the dynamic stimulus. The former measure, though not standard, might have been a fairer reflection of the amount of visual attention assigned to the stimulus, given the dynamic nature of the primary task stimuli that were likely related to many smooth-pursuit movements. Perhaps because of that the difference between semi-predictable and random dot trajectories was only obtained using the former measure. This effect is additionally confirmed by the measurement of the number of saccades to the dynamic stimulus. Predictable dot trajectory was related to a smaller proportion of saccades than the unpredictable trajectories- this effect is very strong. Additionally, a fine-grained analysis of the types of saccades revealed that this was mainly due to the decrease in the number of saccades within the dynamic stimulus. At the same time, the there was an increase in the number of saccades within the static stimulus and between the stimuli in the predictable dot condition, compared to the semi-predictable and random dot conditions. This suggests that dot predictability allowed a transfer of attention from the dynamic to the static stimulus.

However, surprisingly there is also a small, but significant increase in the number of saccades to the dynamic stimulus in the semi-predictable condition, compared to the random condition. The analysis of saccade types revealed that this effect appears only in case of saccades within the dynamic stimulus. This suggests that continuous dot-trajectory in the semi-predictable condition might have been easier to track than discontinuous trajectory in the random condition. In other words, participants might have stopped attempting to track each dot position in the random condition, instead simply fixated on the dynamic side of the screen. It is possible that there was not enough time to plan and execute the next saccade before the dot moved to another location, so there was little benefit in doing so. The signal detection analysis of the symbol detection task showed significantly lower sensitivity when the dot trajectory was random, compared to the other two conditions. Given the significantly more conservative criterion in the random dot condition, we can conclude that the decrease in sensitivity was mainly caused by an increase in misses. That suggests that the task of monitoring the dot for the appearance of their assigned symbol was more difficult when the dot trajectory was random, compared to the other two conditions, which could also lead to a slight decrease in the number of saccades.

There is another problematic aspect of the random trajectory condition. Multiple studies report “onset primacy”, the phenomenon of attentional capture by stimulus onset [5,42–44]. This may be significant in case of the random stimuli, where lack of continuity between successive presentations of the dot may lead to each being treated as a new stimulus. As a result, in the random condition each appearance of the dot may capture more attention by virtue of being perceived as the onset of a new stimulus. In contrast, in the semi-predictable and predictable conditions, dynamic stimuli, because of the continuity of the dot trajectory, would not attract additional attention related to onset primacy.

However, the pattern of differences between the semi-predictable and predictable conditions is very similar to the pattern of differences between the random and predictable conditions. For this reason, difficulty in tracking or presence of multiple onset are unlikely to be the cause behind the observed patterns of results.

What is important, these differences in the allocation of time spent looking at each stimulus were also reflected in the accuracy in the memory test. Static stimuli accompanied by dot moving with a predictable trajectory were recognized in the memory test with significantly higher accuracy than static stimuli accompanied by the less predictably moving dots. That means that when the primary task was more predictable, the additional attention allocated to the secondary task was used in the processing of the static stimulus, leading to better encoding and subsequent improved memory. This demonstrates that shifts of attention observable in the eye-movement patterns represent changes in allocation of a valuable but limited resource that determines which information are processed and subsequently survive in the brain as memories. Utilizing statistical regularities in the environment helps to allocate attention more optimally and improves performance [45–47]. In addition to these findings, this study demonstrates, that predictability of some elements of the environment not only lead to more effective processing of that element, but also offloads the resources to other, less regular or predictable elements of the environment, resulting in a measurable benefit to their processing.

### The role of exogenous attention

The effect of stimulus type (neutral versus social or threatening, depending on the block) was strong and significant for all eye-movements measures in the study: that is, time spent looking at the dynamic stimulus and the proportion of saccades to the dynamic stimulus. Static stimuli “special” from the evolutionary point of view attracted a larger share of visual attention than neutral stimuli, and decreased the amount of attention allocated to the dynamic stimulus. This effect was stronger for threatening stimuli than for social stimuli in case of both the proportion of duration of fixation and proportion of looking time. However, the reason for this difference is unclear—it could be that threatening stimuli have priority over social stimuli in capturing attention, but it could also be due to differences in valence and arousal between these two classes of stimuli.

Additionally, for these measures there was also a significant interaction between stimulus type and block, implying that the difference between neutral and non-neutral stimuli was larger in the threat block compared to the social block. In other words, both threatening and social stimuli were capable of capturing a larger share of attention than neutral stimuli, but this effect was larger for the threatening stimuli. There was no significant difference in the effect strength between threatening and social blocks in case of the proportion of the number of saccades. There was also no difference between the social and threat block in performance in the memory test. However, the memory test involved only neutral stimuli, so we did not expect any differences between the two blocks.

To summarize, the study demonstrated the ability of stimuli with special evolutionary value (such as social or threatening stimuli) to capture attention and effectively distort the allocation of attention that was optimized for another, concurrent task. Similarly, Nummenmaa et al. [12] demonstrated that emotional stimuli were more likely to attract first fixations and be inspected for a longer time than simultaneously presented neutral stimuli.

Unfortunately, the design of the study did not allow us to find out whether this distortion of attention allocation resulted in either worse performance in the symbol detection task for dynamic stimuli accompanied by non-neutral static stimuli, or improved memory for non-neutral stimuli. However, for example Van Damme et al. [9] demonstrated that presence of a threat cue decreased accuracy of target detection, which suggests difficulty with disengaging attention from threats. Similarly, emotionally charged words in a Stroop task interfered with colour naming by increasing the share of attention allocated to the meaning of the word [48].

To sum up, results obtained in this study demonstrate that attention allocation is simultaneously shaped by multiple processes, competing for shares in the same pool of resources. Sudden appearance of a stimulus that is task-irrelevant but important from the evolutionary point of view can distort the allocation of resources optimized to the task at hand.

### Conclusion

This study provides further evidence on the role of predictions in shaping visual attention.

As traditionally conceived, allocation of attention is managed endogenously, via motivation and task-relevance assessment, and exogenously, via factors intrinsic to the stimulus such as its salience and evolutionary importance. However, as this study demonstrates, the attentional resources are also assigned in accordance to stimulus predictability- with predictable stimuli receiving a smaller share of attention than unpredictable ones. This constitutes a “saving” which can be used in processing of concurrent stimuli. Our study shows that such attentional “savings” lead to a measurable benefit in processing and encoding of concurrent stimuli, reflected in improved memory. Therefore, utilizing predictability inherent in the environment saves attentional resources and leads to a “trickle-down” effect, i.e. benefits the processing of other concurrent stimuli.

## Supporting information

S1 Table List of photographs from the International Affective Picture System [40], that were used in the study. (DOCX)

S1 Dataset Proportion of duration of fixations on the dynamic stimulus depending on the task, stimulus type and dot trajectory. Soc- social task, thr—threat task, neu- neutral stimulus, non- non-neutral stimulus, ran—random trajectory, semi- semi-predictable trajectory, pred- predictable trajectory.
(CSV)

S2 Dataset Proportion of time spent looking at the dynamic stimulus depending on the task, stimulus type and dot trajectory. Soc- social task, thr—threat task, neu- neutral stimulus, non- non-neutral stimulus, ran—random trajectory, semi- semi-predictable trajectory, pred- predictable trajectory.
(CSV)

S3 Dataset Proportion of the number of saccades to the dynamic stimulus depending on the task, stimulus type and dot trajectory. Soc- social task, thr—threat task, neu- neutral stimulus, non- non-neutral stimulus, ran—random trajectory, semi- semi-predictable trajectory, pred- predictable trajectory.
(CSV)

S4 Dataset Loglinear sensitivity (d’) and criterion in the symbol detection task, depending on the dot trajectory. (CSV)

S5 Dataset Loglinear sensitivity (d’) and criterion for each of the memory tasks (social and threat). (CSV)

S6 Dataset Accuracy in the memory test depending on the dot trajectory. (CSV)

S1 Fig A. Sensitivity (d’) and B. Criterion (c) in the symbol detection task, depending on the dot trajectory. Error bars denote within-subjects 95% confidence intervals.
(EPS)

S2 Fig The proportion of duration of fixations on the dynamic stimulus, separately for the neutral and non-neutral (social or threat, depending on the block) stimuli. Error bars denote within-subjects 95% confidence intervals.
(EPS)

S3 Fig Histograms of saccade length for each level of dot trajectory predictability. (PDF)

S1 Appendix Proportion of duration of fixations on the dynamic stimulus. (DOCX)
