# Supplementary material for: The trickle-down effect of predictability: Secondary task performance benefits from predictability in the primary task
Source: PLoS One. 2017 Jul 10;12(7):e0180573. doi: 10.1371/journal.pone.0180573 (PMC5503276; doi:10.1371/journal.pone.0180573)
Supplement: S1 Table — (DOCX) [file pone.0180573.s001.docx]

**Table 1. List of photographs from the International Affective Picture System (Lang, Bradley, & Cuthbert, 2008), that were used in the study.**

| **Description** | **No** | **Valence** | **Arousal** | **Complexity** | **Task** | **Stimulus Type** | **Experimental Session** | **Testing Session** |
| --- | --- | --- | --- | --- | --- | --- | --- | --- |
| Butterfly | 1603 | 6.90 | 3.37 | 4.71 | Social | Neutral | No | Foil |
| Grouper | 1910 | 6.71 | 3.29 | 6.06 | Social | Neutral | No | Foil |
| Flower | 5030 | 6.51 | 2.74 | 4.35 | Social | Neutral | No | Foil |
| Galaxy | 5301 | 6.54 | 5.21 | 4.47 | Social | Neutral | No | Foil |
| Mushrooms | 5534 | 4.84 | 3.14 | 4.65 | Social | Neutral | No | Foil |
| Puzzle | 7061 | 5.40 | 3.66 | 4.41 | Social | Neutral | No | Foil |
| Clock | 7211 | 4.81 | 4.20 | 2.94 | Social | Neutral | No | Foil |
| Food | 7481 | 6.53 | 4.92 | 5.94 | Social | Neutral | No | Foil |
| Cabinet | 7705 | 4.77 | 2.65 | 2.75 | Social | Neutral | No | Foil |
| Mountains | 5631 | 7.29 | 3.86 | 3.94 | Threat | Neutral | No | Foil |
| Flowers | 5811 | 7.23 | 3.30 | 6.18 | Threat | Neutral | No | Foil |
| Clouds | 5870 | 6.78 | 3.10 | 3.41 | Threat | Neutral | No | Foil |
| Wines | 7280 | 7.20 | 4.46 | 5.35 | Threat | Neutral | No | Foil |
| Pizza | 7350 | 7.10 | 4.97 | 5.06 | Threat | Neutral | No | Foil |
| Hamburger | 7451 | 6.68 | 5.84 | 3.53 | Threat | Neutral | No | Foil |
| Grapes | 7472 | 6.25 | 4.00 | 6.53 | Threat | Neutral | No | Foil |
| Ferry | 7492 | 7.41 | 4.91 | 5.71 | Threat | Neutral | No | Foil |
| Money | 8501 | 7.91 | 6.44 | 4.75 | Threat | Neutral | No | Foil |
| Butterfly | 1605 | 6.59 | 3.43 | 5.88 | Social | Neutral | Yes | Target |
| Fish | 1900 | 6.65 | 3.46 | 7.12 | Social | Neutral | Yes | Target |
| Flower | 5010 | 7.14 | 3.00 | 3.69 | Social | Neutral | Yes | Target |
| Galaxy | 5300 | 6.91 | 4.36 | 4.35 | Social | Neutral | Yes | Target |
| Mushroom | 5500 | 5.42 | 3.00 | 4.06 | Social | Neutral | Yes | Target |
| Coffeecup | 7057 | 5.35 | 3.39 | 5.06 | Social | Neutral | Yes | Target |
| Clock | 7190 | 5.55 | 3.84 | 3.71 | Social | Neutral | Yes | Target |
| FileCabinets | 7224 | 4.45 | 2.81 | 4.00 | Social | Neutral | Yes | Target |
| Turkey | 7230 | 7.38 | 5.52 | 5.82 | Social | Neutral | Yes | Target |
| Parrots | 1333 | 6.11 | 3.17 | 4.88 | Social | Neutral | Yes | No |
| Flower | 5020 | 6.32 | 2.63 | 4.71 | Social | Neutral | Yes | No |
| Garden | 5202 | 7.25 | 3.73 | 4.71 | Social | Neutral | Yes | No |
| Boat | 5390 | 5.59 | 2.88 | 4.47 | Social | Neutral | Yes | No |
| Boat | 5395 | 5.34 | 4.23 | 5.06 | Social | Neutral | Yes | No |
| Grain | 5726 | 6.23 | 2.84 | 4.41 | Social | Neutral | Yes | No |
| Flowers | 5731 | 5.39 | 2.74 | 5.35 | Social | Neutral | Yes | No |
| Mountain | 5814 | 7.15 | 4.82 | 5.29 | Social | Neutral | Yes | No |
| Train | 7033 | 5.40 | 3.99 | 6.59 | Social | Neutral | Yes | No |
| Shipyard | 7036 | 4.88 | 3.32 | 7.00 | Social | Neutral | Yes | No |
| Luggage | 7081 | 5.36 | 3.96 | 5.29 | Social | Neutral | Yes | No |
| Scarves | 7205 | 5.56 | 2.93 | 4.41 | Social | Neutral | Yes | No |
| Building | 7242 | 5.28 | 3.83 | 7.12 | Social | Neutral | Yes | No |
| Cupcakes | 7405 | 7.38 | 6.28 | 4.00 | Social | Neutral | Yes | No |
| Candy | 7410 | 6.91 | 4.55 | 4.59 | Social | Neutral | Yes | No |
| FerrisWheel | 7508 | 7.02 | 5.09 | 4.82 | Social | Neutral | Yes | No |
| House | 7530 | 6.71 | 4.00 | 4.65 | Social | Neutral | Yes | No |
| Wingwalker | 8341 | 6.25 | 6.40 | 6.25 | Social | Neutral | Yes | No |
| AttractiveFem | 2019 | 6.07 | 4.31 | 4.94 | Social | Non-neutral | Yes | No |
| Kid | 2035 | 7.52 | 3.69 | 5.47 | Social | Non-neutral | Yes | No |
| Baby | 2040 | 8.17 | 4.64 | 5.12 | Social | Non-neutral | Yes | No |
| Baby | 2075 | 7.32 | 5.27 | 5.19 | Social | Non-neutral | Yes | No |
| Family | 2156 | 7.12 | 4.34 | 5.53 | Social | Non-neutral | Yes | No |
| NeutMan | 2215 | 4.63 | 3.38 | 3.24 | Social | Non-neutral | Yes | No |
| Class | 2217 | 6.24 | 4.08 | 6.12 | Social | Non-neutral | Yes | No |
| BoysReading | 2222 | 7.11 | 4.08 | 4.47 | Social | Non-neutral | Yes | No |
| ChildCamera | 2302 | 6.43 | 3.64 | 4.53 | Social | Non-neutral | Yes | No |
| Binoculars | 2314 | 7.55 | 4.00 | 4.76 | Social | Non-neutral | Yes | No |
| Father | 2339 | 6.72 | 4.16 | 4.94 | Social | Non-neutral | Yes | No |
| Children | 2341 | 7.38 | 4.11 | 5.41 | Social | Non-neutral | Yes | No |
| Children | 2342 | 6.20 | 4.06 | 7.18 | Social | Non-neutral | Yes | No |
| Mother/Child | 2359 | 5.87 | 3.94 | 5.24 | Social | Non-neutral | Yes | No |
| Reading | 2377 | 5.19 | 3.50 | 6.00 | Social | Non-neutral | Yes | No |
| Artist | 2382 | 5.67 | 3.75 | 5.82 | Social | Non-neutral | Yes | No |
| Secretary | 2383 | 4.72 | 3.41 | 4.71 | Social | Non-neutral | Yes | No |
| Couple | 2390 | 5.40 | 3.57 | 4.94 | Social | Non-neutral | Yes | No |
| ManW/Fish | 2392 | 6.15 | 3.85 | 5.12 | Social | Non-neutral | Yes | No |
| Mom/Son | 2435 | 5.84 | 3.94 | 6.71 | Social | Non-neutral | Yes | No |
| Musician | 2488 | 5.73 | 3.91 | 5.94 | Social | Non-neutral | Yes | No |
| Woman | 2513 | 5.80 | 3.29 | 4.41 | Social | Non-neutral | Yes | No |
| ManW/Dog | 2521 | 5.78 | 4.10 | 5.06 | Social | Non-neutral | Yes | No |
| Women | 2595 | 4.88 | 3.71 | 5.18 | Social | Non-neutral | Yes | No |
| Baby | 2660 | 7.75 | 4.44 | 5.00 | Social | Non-neutral | Yes | No |
| Chess | 2840 | 4.91 | 2.43 | 5.06 | Social | Non-neutral | Yes | No |
| Watermelon | 7325 | 7.06 | 3.55 | 4.82 | Social | Non-neutral | Yes | No |
| Clouds | 5551 | 7.31 | 3.26 | 4.06 | Threat | Neutral | Yes | Target |
| Mountains | 5600 | 7.57 | 5.19 | 5.41 | Threat | Neutral | Yes | Target |
| Nature | 5760 | 8.05 | 3.22 | 5.53 | Threat | Neutral | Yes | Target |
| Alcohol | 7279 | 6.22 | 5.19 | 5.69 | Threat | Neutral | Yes | Target |
| Desserts | 7320 | 6.54 | 4.44 | 4.71 | Threat | Neutral | Yes | Target |
| Pizza | 7351 | 5.82 | 4.25 | 4.29 | Threat | Neutral | Yes | Target |
| Cheeseburger | 7450 | 6.40 | 5.05 | 3.24 | Threat | Neutral | Yes | Target |
| Ferrry | 7489 | 6.54 | 4.49 | 6.76 | Threat | Neutral | Yes | Target |
| Money | 8502 | 7.51 | 5.78 | 4.00 | Threat | Neutral | Yes | Target |
| Butterfly | 1602 | 6.50 | 3.43 | 4.76 | Threat | Neutral | Yes | No |
| Shrimp | 1903 | 5.50 | 4.25 | 4.18 | Threat | Neutral | Yes | No |
| Seaside | 5210 | 8.03 | 4.60 | 5.82 | Threat | Neutral | Yes | No |
| Liftoff | 5450 | 7.01 | 5.84 | 5.76 | Threat | Neutral | Yes | No |
| Fireworks | 5480 | 7.53 | 5.48 | 4.76 | Threat | Neutral | Yes | No |
| Shark | 5622 | 6.33 | 5.34 | 5.06 | Threat | Neutral | Yes | No |
| WinterStreet | 5635 | 6.25 | 3.97 | 6.06 | Threat | Neutral | Yes | No |
| Beach | 5833 | 8.22 | 5.71 | 6.47 | Threat | Neutral | Yes | No |
| Flowers | 5849 | 6.65 | 4.89 | 6.59 | Threat | Neutral | Yes | No |
| Earth | 5890 | 6.67 | 4.60 | 4.59 | Threat | Neutral | Yes | No |
| Buttons | 7001 | 5.32 | 3.20 | 5.71 | Threat | Neutral | Yes | No |
| Tools | 7019 | 5.20 | 3.36 | 4.63 | Threat | Neutral | Yes | No |
| Peanuts | 7300 | 5.64 | 3.25 | 3.71 | Threat | Neutral | Yes | No |
| Garlic | 7354 | 5.51 | 3.73 | 3.76 | Threat | Neutral | Yes | No |
| Skyline | 7570 | 6.97 | 5.54 | 5.12 | Threat | Neutral | Yes | No |
| Traffic | 7590 | 4.75 | 3.80 | 5.13 | Threat | Neutral | Yes | No |
| City | 7650 | 6.62 | 6.15 | 7.13 | Threat | Neutral | Yes | No |
| SportCar | 8510 | 7.32 | 4.93 | 4.88 | Threat | Neutral | Yes | No |
| Snake | 1050 | 3.46 | 6.87 | 3.75 | Threat | Non-neutral | Yes | No |
| Snake | 1120 | 3.79 | 6.93 | 4.88 | Threat | Non-neutral | Yes | No |
| Spider | 1202 | 3.35 | 5.94 | 6.18 | Threat | Non-neutral | Yes | No |
| Spider | 1205 | 3.65 | 5.79 | 7.06 | Threat | Non-neutral | Yes | No |
| Roach | 1270 | 3.68 | 4.77 | 5.00 | Threat | Non-neutral | Yes | No |
| Roaches | 1271 | 3.19 | 5.37 | 6.24 | Threat | Non-neutral | Yes | No |
| Rat | 1280 | 3.66 | 4.93 | 6.76 | Threat | Non-neutral | Yes | No |
| PitBull | 1300 | 3.55 | 6.79 | 5.47 | Threat | Non-neutral | Yes | No |
| AttackDog | 1304 | 3.37 | 6.37 | 4.65 | Threat | Non-neutral | Yes | No |
| Attack | 3500 | 2.21 | 6.99 | 5.53 | Threat | Non-neutral | Yes | No |
| Injury | 3550 | 2.54 | 5.92 | 5.18 | Threat | Non-neutral | Yes | No |
| Aimedgun | 6190 | 3.57 | 5.64 | 4.35 | Threat | Non-neutral | Yes | No |
| Abduction | 6312 | 2.48 | 6.37 | 5.12 | Threat | Non-neutral | Yes | No |
| Attack | 6313 | 1.98 | 6.94 | 5.24 | Threat | Non-neutral | Yes | No |
| BeatenFem | 6315 | 2.31 | 6.38 | 5.53 | Threat | Non-neutral | Yes | No |
| Attack | 6350 | 1.90 | 7.29 | 4.41 | Threat | Non-neutral | Yes | No |
| Attack | 6370 | 2.70 | 6.44 | 3.59 | Threat | Non-neutral | Yes | No |
| Attack | 6510 | 2.46 | 6.96 | 4.18 | Threat | Non-neutral | Yes | No |
| Attack | 6520 | 1.94 | 6.59 | 5.18 | Threat | Non-neutral | Yes | No |
| Attack | 6540 | 2.19 | 6.83 | 5.18 | Threat | Non-neutral | Yes | No |
| Attack | 6560 | 2.16 | 6.53 | 5.00 | Threat | Non-neutral | Yes | No |
| CarTheft | 6571 | 2.85 | 5.59 | 5.18 | Threat | Non-neutral | Yes | No |
| PlaneCrash | 9050 | 2.43 | 6.36 | 7.44 | Threat | Non-neutral | Yes | No |
| Execution | 9414 | 2.06 | 6.49 | 6.19 | Threat | Non-neutral | Yes | No |
| DentalExam | 9584 | 3.34 | 4.96 | 5.31 | Threat | Non-neutral | Yes | No |
| Injecting | 9590 | 3.08 | 5.41 | 4.50 | Threat | Non-neutral | Yes | No |
| Injection | 9592 | 3.34 | 5.23 | 5.19 | Threat | Non-neutral | Yes | No |

Lang, P. J., Bradley, M. M., & Cuthbert, B. N. (2008). *International affective picture system (IAPS): Affective ratings of pictures and instruction manual. Technical Report A-8*. Gainesville, FL.
