# Supplementary material for: The trickle-down effect of predictability: Secondary task performance benefits from predictability in the primary task
Source: PLoS One. 2017 Jul 10;12(7):e0180573. doi: 10.1371/journal.pone.0180573 (PMC5503276; doi:10.1371/journal.pone.0180573)
Supplement: S3 Fig — (PDF) [file pone.0180573.s010.pdf]

Histograms of saccade lenght for each level of dot trajectory predictability

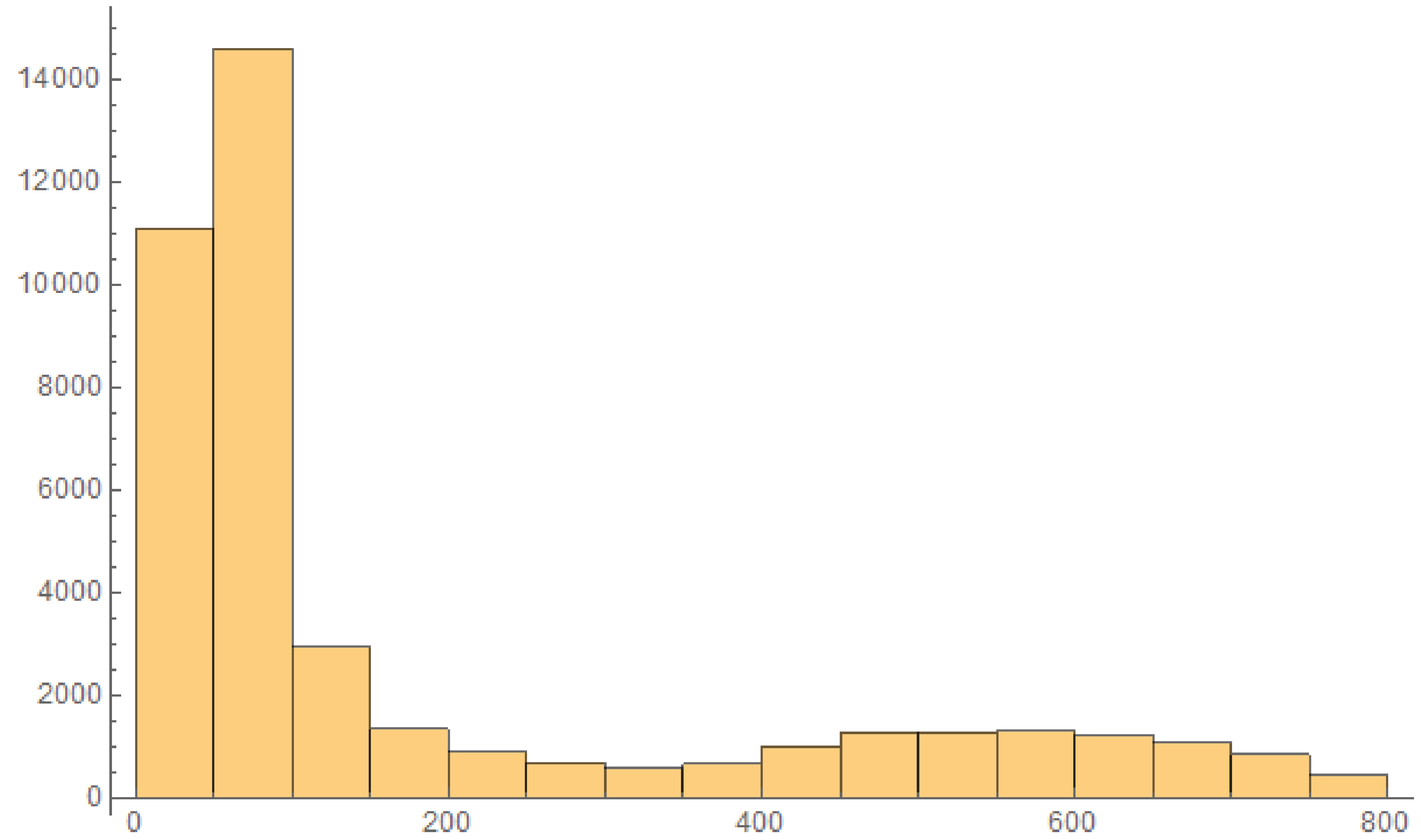

A. Predictable dot trajectory

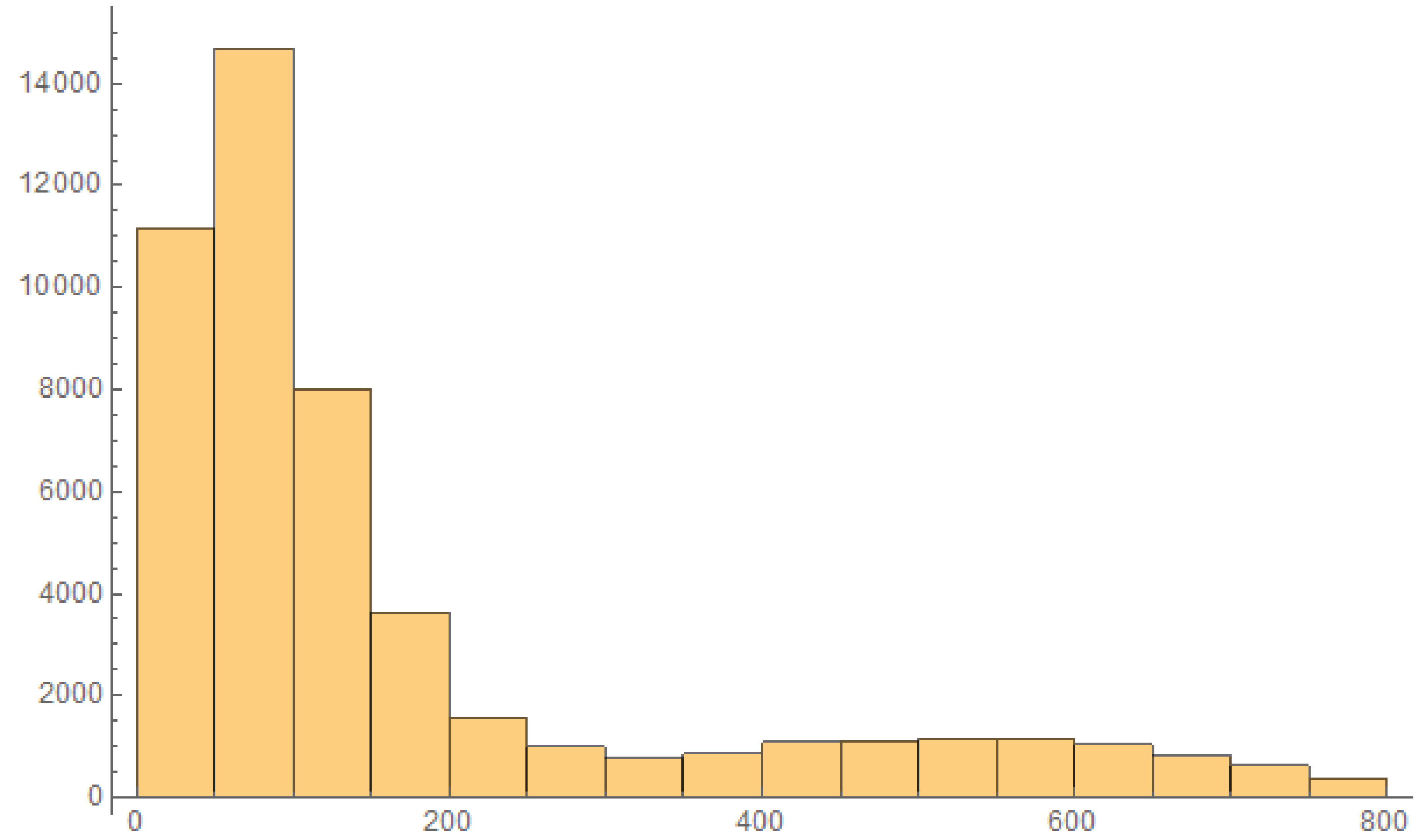

B. Semi- predictable dot trajectory

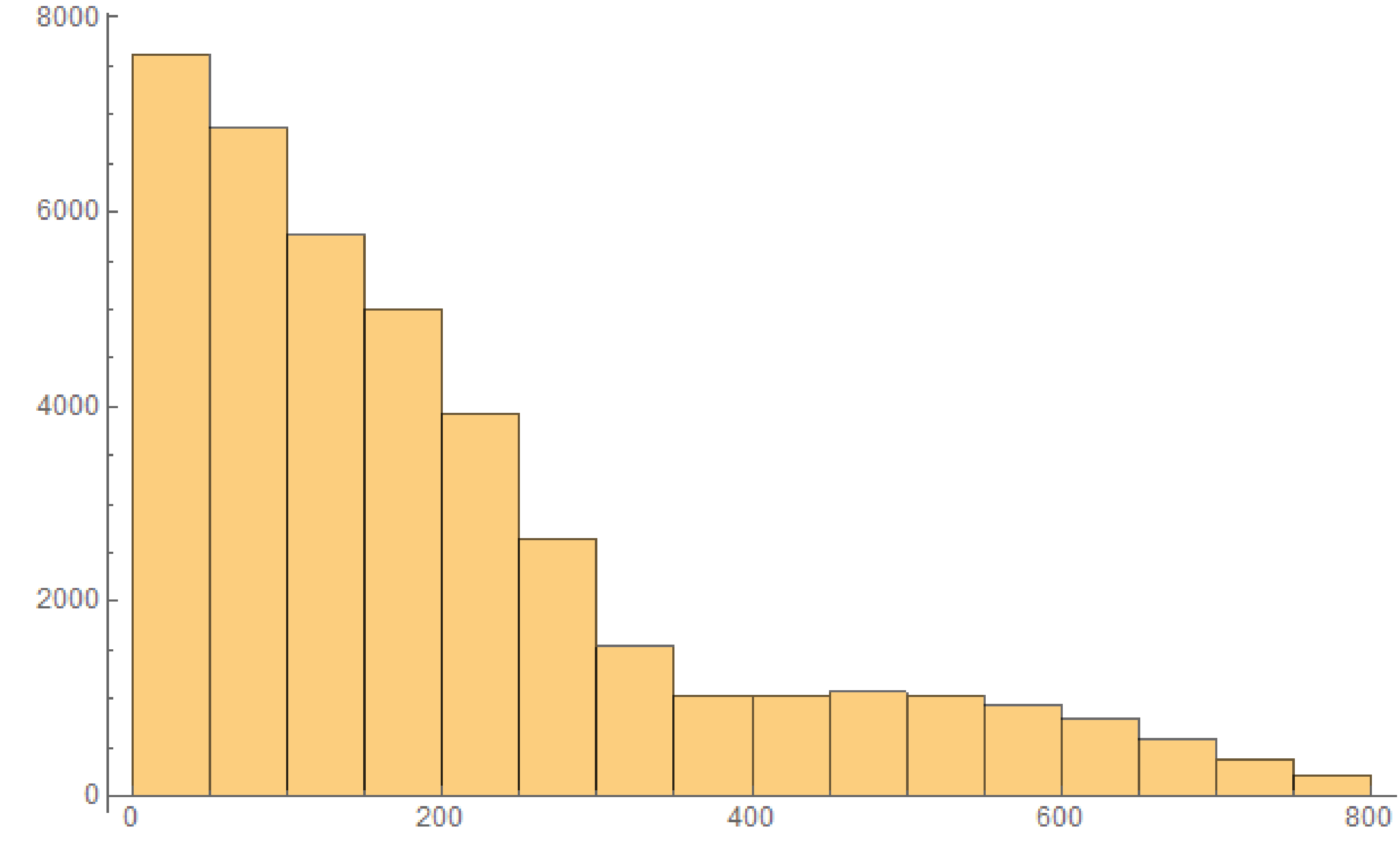

C. Random dot trajectory
