# Supplementary material for: The trickle-down effect of predictability: Secondary task performance benefits from predictability in the primary task
Source: PLoS One. 2017 Jul 10;12(7):e0180573. doi: 10.1371/journal.pone.0180573 (PMC5503276; doi:10.1371/journal.pone.0180573)
Supplement: S1 Appendix — (DOCX) [file pone.0180573.s011.docx]

## S9 Supporting Information

### Proportion of duration of fixations on the dynamic stimulus

For each trial, we calculated the total duration of fixations positioned within each AOI. Additionally, we calculated the proportional duration of fixations on the dynamic stimulus (duration of fixations henceforth) by dividing the total duration of fixations on the dynamic stimulus by the total duration of all fixations in the trial.

We subjected the duration of fixations on the dot side to a repeated-measures 2 (block: social vs. threat) x 2 (static stimulus type: neutral vs. non-neutral, i.e. either social or threatening, depending on the block) x 3 (dot trajectory: random, semi-predictable, predictable) ANOVA (Fig S10). Greenhouse- Geisser correction was used to adjust the degrees of freedom, when the sphericity assumption was violated.


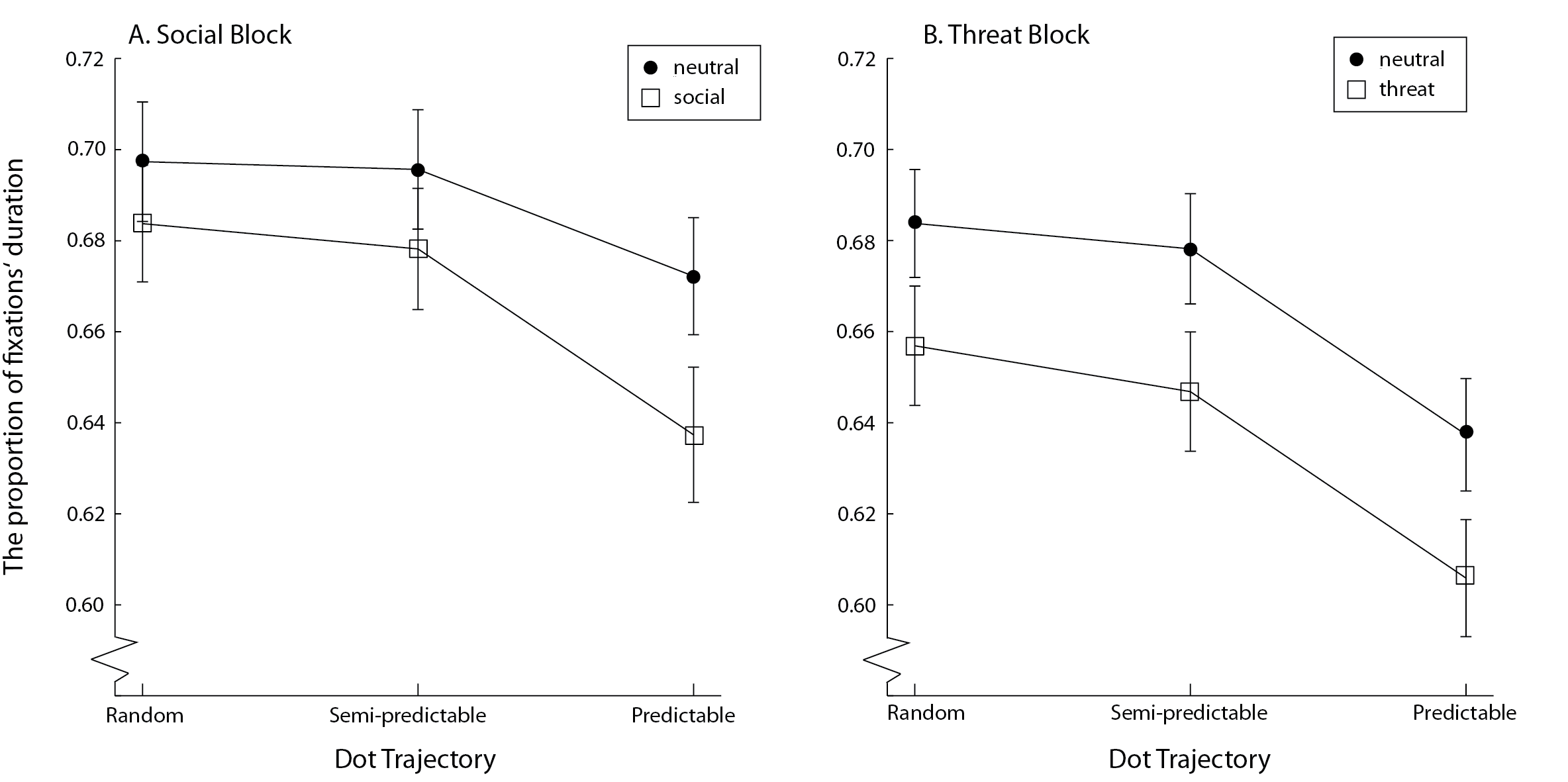


**Fig S10. The proportion of duration of fixations on the dynamic stimulus, separately for the neutral and non-neutral (social or threat, depending on the block) stimuli. Error bars denote SEM.**

Participants spent more time looking at the dynamic stimulus in the social block than in the threat block, F(1,132)=3.95, p=.05, η_p_²= .03. Participants spent more time looking at the dynamic stimulus when the accompanying static stimulus was neutral, than when it was not neutral, F(1,132)=87.4, p<.001, η_p_²=.40. Finally, there was a significant difference in the proportion of fixations on the dynamic stimulus depending on the dot trajectory, F(1.87, 246.3)=46.57, p<.001, η_p_²=.26. Contrasts revealed that participants looked significantly less at the dynamic stimulus when the dot trajectory was predictable compared to when it was semi-predictable, F(1,132)=78.93, p<.001, η_p_²=.37. However, there was no significant difference between the semi-predictable and random conditions, F(1,132)=1.61, p=.21, η_p_²=.01.

There was a significant interaction between block and stimulus type, F(1,132)=9.31, p=.01 η_p_²=.07, implying that the difference between the neutral and non-neutral stimuli was larger in the threat block, compared to the social block. The interaction between block and dot trajectory was not significant, F(2,264)=1.60, p=.20, η_p_²=.01, and neither was the interaction between stimulus type and dot trajectory, F(2,264)=1.04, p=.35, η_p_²=.01. Finally, the three-way interaction between block, stimulus type and dot trajectory was also non-significant, F(2,264)=0.98, p=.38, η_p_²=.01.
